# Smart cranioplasty in cleidocranial dysplasia: bridging robotics and neurotechnology for precision reconstruction

**DOI:** 10.1097/MS9.0000000000004359

**Published:** 2025-11-14

**Authors:** Duaa Seengar, Hamza Ali, Muhammad Talha, Mahnoor Fatima, Ubaid Ur Rehman

**Affiliations:** a Department of Medicine, Jinnah Sindh Medical University, Karachi, Pakistan; b Department of Medicine, Karachi medical and Dental college, Karachi, Pakistan; c Department of Medicine, King Edward Medical University, Lahore, Pakistan; d Department of Medicine, Shaheed Ziaur Rahman Medical College, Rajshahi Medical University, Bogura, Bangladesh

*To the Editor,*

Cleidocranial dysplasia (CCD) is a congenital skeletal dysplasia characterized by hypoplasia or agenesis of the clavicle, brachycephalic skull, midfacial hypoplasia, prolonged patency of the fontanelles, and mild to moderate growth retardation. CCD is an autosomal dominant disorder with a prevalence of about 1/1 000 000 globally. RUNX2 (runt-related transcription factor 2 gene) is a key regulator of osteogenesis, situated on chromosome 6 (6p21). The mutation in the RUNX2 leads to cleidocranial dystosis. Because of impaired bone ossification of the contours of the embryonic vertebral arch, spinal abnormalities include spina bifida, scoliosis, kyphosis/kyphoscoliosis, spondylolysis, spondylolisthesis, and hemivertebra^[1]^. Surgical correction may be undertaken for cleidocranial hypoplasia, while calcium and vitamin D supplementation are recommended for reduced bone density^[2]^. This is in line with the TITAN Guidelines on the need for transparency in AI use in healthcare^[3]^.

Delayed cranioplasty causes major consequences. Therefore, single-stage cranioplasty, which primarily restores the skull defect with a patient-specific prefabricated cranial implant CCI, is more widely adopted. The robotic system offers precise CCI modification for single-stage cranioplasty and enhanced clinical implementation by addressing challenges in patients’ CT, optimizing CCI resizing algorithms, and advancing surgical automation. Furthermore, the robotic system provides quicker and more accurate CCI modifications^[4]^. Neurotechnology plays a pivotal role in integrating brain–machine interfaces, biosensors, and adaptive neural monitoring systems within these cranial constructs, bridging surgery with digital neuroscience^[5]^. Moreover, CCI integrates implanted neurotechnology, such as ventriculostomy shunts, intracranial pressure monitoring devices, and drug delivery systems, transforming from “basic” implants into “smart” implants with advanced functionality. These smart implants optimize the patients’ outcomes by utilizing the cranial space to build advanced technology^[6]^.

Recent studies suggest that cranioplasty results depend on timing and patient factors. A review of 754 cases showed very low mortality but a notable complication rate that highlights its potential applications. Early surgeries were linked to higher infection and hydrocephalus risks. Operations done between 15 and 30 days had fewer bone resorption issues, and older patients showed better outcomes with lower resorption^[7]^. While another study reported a relatively low complication rate of 15.6% consistent with previous research, it also recorded a low infection rate of 3.9% indicating safe surgical outcomes. The infection rate with autologous bone was comparable to synthetic materials, showing good reliability. The study also notes that titanium implants are more prone to wound complications and long-term scalp thinning issues. Overall, the findings support cranioplasty as an effective and safe reconstruction^[8]^.

The use of advanced smart cranioplasty techniques in patients with cleidocranial dysplasia for cranial defect repair and osseointegration promotion has been innovative, but there are some limitations and shortcomings. The procedure leads to the general cranioplasty risks, which are some of the most common ones, such as infections, hematomas, and bone flap resorption, depending on a cohort from Brunei where 17.6% of the instances required reoperation due to adverse events^[7]^. Moreover, the question of when is the best period for the intervention is still a matter of controversy; cranioplasty done early (<3 months postcraniectomy) has been linked to the taking place of infection and hydrocephalus whereas waiting for more than 6–12 months is likely to cause the patient to suffer from seizures and neurological decline, which is the outcome of a huge single-center retrospective study with 754 cases over a decade^[8]^. In the case of cleidocranial dysplasia, the literature does not cover the syndrome-related aspects, such as the RUNX2 mutations that affect bone healing or the use of “smart” biomaterials (e.g., 3D-printed scaffolds with bioactive coatings) in this genetic background, therefore, pointing out a critical evidence gap for customized outcomes, long-lasting effects, and pediatric considerations in dysplastic crania^[7,8]^.

The use of robotic-assisted single-stage cranioplasty with patient-specific implants (CCI) in the case of cleidocranial dysplasia allows for resizing with precision and quick intraoperative adaptation through the CT-guided process, and converting standard implants into smart platforms that integrate neurotechnology like ICP monitors and drug-delivery systems^[4,6]^. One of the main benefits of this advancement is the reduction of the chances of delayed cranioplasty, while at the same time coping with the abovementioned CCD-related problems like ongoing fontanelles and midfacial hypoplasia^[1,2]^. A recent review of the literature points out the trend of various complication rates (3.9–15.6%) depending on a number of factors such as timing, material, and technique, but no long-term data regarding resorption and cost-effectiveness are available^[7,8]^. One of the next steps would be the creation of multicenter registries with implantation follow-up over 10 years to monitor the durability of the implants, as well as radiological scoring for osseointegration and randomized trials comparing titanium, autologous, and bioresorbable CCIs in CCD cohorts and AI-driven predictive modeling for infection risk, and even experimenting with embedded biosensors for real-time cranial compliance monitoring to personalize and advance proactive care^[5–8]^.

In conclusion, the findings suggest that cranioplasty remains a safe and reliable reconstructive option. Both autologous and synthetic materials can produce satisfactory outcomes when used appropriately. Proper technique and patient care can reduce the risks. The overall findings emphasize that technique, material choice, and postoperative care strongly influence results. Continued research and better follow-up can further improve the safety and success.

## Data Availability

Not applicable to this study.
